# Therapeutic Effects of Anthocyanins for Vision and Eye Health

**DOI:** 10.3390/molecules24183311

**Published:** 2019-09-11

**Authors:** Yuri Nomi, Keiko Iwasaki-Kurashige, Hitoshi Matsumoto

**Affiliations:** 1 Niigata University of Pharmacy and Applied Life Sciences, 265-1 Higashijima, Akiha-ku, Niigata 956-8603, Japan; ynomi@nupals.ac.jp; 2 Functional Material Division, Meiji Food Materia Co., Ltd, 4–16, Kyobashi 2-chome, Chuo-ku, Tokyo 104-0031, Japan; keiko.kurashige@meiji.com

**Keywords:** anthocyanin, myopia, glaucoma, ciliary muscle, blood circulation

## Abstract

Anthocyanin (AC) is widely used as supplement of eye health in Europe and in East Asia. In this review, I describe AC effects to clarify the mechanism is important in order to understand the effects of AC on vision health. The bioavailability of AC is quite low but, reported as intact form and many kinds of metabolite. And AC passes through the blood-aqueous fluid barrier and blood-retinal barrier. In vitro study, AC had a relaxing effect on ciliary muscle which is important to treat both myopia and glaucoma. And AC stimulate the regeneration of rhodopsin in frog rod outer segment. Furthermore, AC could inhibit the axial length and ocular length elongation in a negative lens-induced chick myopia model. In addition, we summarized clinical studies of AC intake improved dark adaptation and transient myopic shift and the improvement on retinal blood circulation in normal tension glaucoma patients.

## 1. Introduction

During the last decade the rapid growth of smartphone, computer and video display terminal (VDT) use in the office and private place has led to an increase in ocular problems including eye discomfort, blurred vision, eye strain, eye pain and visual fatigue [1,2]. In response to an aging population in developed countries, rates of ocular disease associated with eye strain and external factors such as blue light and UV have increased.

An anthocyanin (AC) complex from bilberry (*Vaccinium myrtillus* L.) fruit is widely used in Europe for medicinal purposes [3] and as a dietary supplement in countries in East Asia, especially Japan [4]. Bilberry AC has been reported to enhance night visual acuity [5] and is used as a supplement to improve vision health [3].

Consumers have access to a wide range of methods and treatments to mitigate the effects ophthalmologic disorders. However, whether AC consumption can improve ophthalmologic health is unclear. Ophthalmologic diseases often have a variety of symptoms, and a single supplement such as AC is unlikely to produce improvement in all ophthalmologic disorders. Thus, clarification of the mechanisms by which AC could improve vision health and provide beneficial effects to lessen the symptoms of ophthalmologic disorders such as asthenopia, myopia and glaucoma is needed. Canter and Ernst reviewed clinical studies that focused on the use of bilberry AC supplementation to improve night vision by, but the results for these studies were negative [6]. Kalt et al. also reviewed studies that focused on the polyphenol’s effects on vision health [7] including in vitro researches demonstrating that ACs and other flavonoids interact with rhodopsin to modulate visual transductional function, although the relationship between the mechanism of action and clinical benefits of these agents remains unclear. Accordingly, we began to evaluate the physiological effects of AC in the context of vision health.

**Figure 1:**
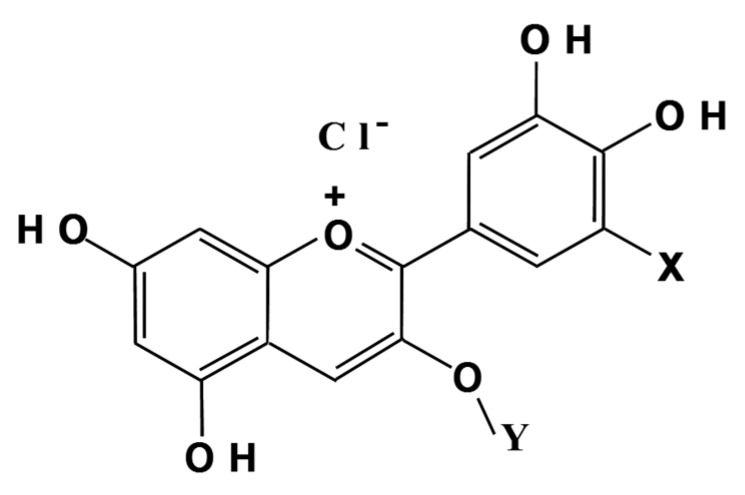
Structure of anthocyanin in blackcurrants. The chemical structure of each of the four anthocyanins (ACs) is as follows: D3R (X = OH, Y = glucosylrhamnose), D3G (X = OH, Y = glucose), C3R (X = H, Y = glucosylrhamnose) and C3G (X = H, Y = glucose).

There are multiple types of AC high content fruits and vegetables, wherein varieties of AC differ depending on the food’s origin. In our research we focused on AC from blackcurrant fruits (*Ribes nigrum* L.), which are rich in AC and are commonly consumed worldwide. With only four different AC, the AC composition of blackcurrants is simpler than that for bilberries, which have fifteen AC components (Figure 1). The typical profile of blackcurrant anthocyanin (BCA) is 47% delphinidin-3-rutinoside (D3R), 13% delphinidin-3-glucoside (D3G), 35% cyanidin-3-rutinoside (C3R) and 5% cyanidin-3-glucoside (C3G). These four ACs have been purified and crystallized [8].

This review covers the published and unpublished work concerning the effect of AC on vision health from our and other groups. We reported that D3R has a relaxing effect after Endothelin-1 induced contraction on bovine ciliary muscle [9]. One theory for the cause of refractive myopia in childhood is that ciliary muscle becomes spastic as hyper-contraction during close up work (near work), leading to spasmodic refraction of the lens [10]. Extensive studies have been conducted to identify agents that can promote ciliary muscle (CM) relaxation and would be valuable to treat both glaucoma and pseudo myopia [11]. We also reported that cyanidin glycosides stimulate the regeneration of rhodopsin in frog rod outer segment membranes in vitro [12]. In another study we showed that AC treatment decreases axial and ocular length elongation in chicks fitted with negative lens that serves as an animal model of myopia [13,14]. In a series of investigations in a double-masked, placebo-controlled, crossover study on humans we demonstrated that the ingestion of BCA improved dark adaptation and transient myopic shift of refractive status [15]. Meanwhile, Ohguro et al. showed that AC affects the blood flow in retina in normal tension glaucoma patients [16,17].

## 2. BCA Properties, Bioavailability and Distribution in Eye Tissues

There are many reports suggest that AC are absorbed directly (without any metabolization) and then distributed in the blood before being excreted intact into the urine [18,19,20]. BCA consists mainly of AC rutinosides, which have higher bioavailability compared to AC monoglucoside precursors. Indeed, after a 200 mg oral dose of BCA, the total recovery rate of the AC rutinoside in the urine is around 0.1% whereas that of the AC glucoside is only 0.05%. In total, the concentration of BCA components in human plasma reached 35 nmol/L [21]. However, AC degradation products and metabolites are also reported in humans and rats including phenolic acids derivatives (e.g., gallic acid, protocatechuic acid, syringic acid, vanillic acid, caffeic acid, phloroglucinol acid and phloroglucinol aldehyde [22,23].

Furthermore, Czank et al. conducted a study using ^13^C-labeled C3G to examine human metabolism of AC [24]. In this study, eight adult male volunteers received 500 mg ^13^C-labeled C3G and ^13^C levels in blood, urine, feces and breath were measured over the next 48 h. The calculated bioavailability was 12.38% ± 1.38%. They found that ^13^C is excreted both as intact C3G, as well as various conjugates and metabolites such as cyanidin glucuronate conjugate, cyanidin sulfate conjugate, protocatechuic acid, 4-dihydroxyphenylacetic acid, vanillic acid, 4-hydroxyphenylacetic acid, ferulic acid caffeic acid and hippuric acid; the main AC metabolites are hippuric acid and carbon dioxide.

The action of these AC decomposition products and metabolites should be considered in the context of mechanistic effects, particularly since these substances are produced not only from ACs, but also from other polyphenols. We hypothesize that these products including intact AC, represent the physiologically active agents of AC, despite their low level of uptake.

BCA passes intact through the blood-brain barrier and blood-retinal barrier in both rabbits and rats after intravenous and intraperitoneal administration, respectively [25]. In addition, the total concentration of AC in several ocular tissues was higher than that measured in plasma, a finding that suggests that AC can concentrate in ocular tissues. We also found significantly high AC concentrations in some ocular tissues when BCA was administered either intravenously or intraperitoneally (Table 1). In rats given intraperitoneal administration of 108 mg AC/body weight (BW) kg, we showed that intact AC is absorbed and distributed into ocular tissues including the cornea (20.6 μg/g), aqueous humor (6.72 μg/mL), ciliary body and iris (12.9 μg/g), sclera with choroid (245 μg/g) and retina (6.89 μg/g) with a plasma concentration of 2.30 μg/mL. The high AC concentration in the choroid may be due to the propensity of AC to bind proline-rich proteins such as collagen, which is abundant in this tissue [26]. In rabbits, 30 min after intravenous administration of 4.32 mg/BW kg AC were undetectable in the lens, but present in the sclera (3.0 μg/g), choroid (3.0 μg/g), cornea (0.55 μg/g), aqueous humor (1.19 μg/mL), ciliary body (2.04 μg/g), iris (1.11 μg/g) and retina (6.89 μg/g), whereas the plasma concentration was 12.42 μg/mL. The eye tissue distribution of orally administered AC could not be determined due to the low concentrations in both the plasma and whole eyeball (approximately 1 μg/mL and 100 ng/g, respectively). In conclusion, the results show that ACs can pass the blood-retinal barrier after oral administration, but uptake is more effective with intraperitoneal or intravenous delivery.

## 3. Stimulatory on Rhodopsin Regeneration by Cyanidin Glycosides

**Figure 2:**
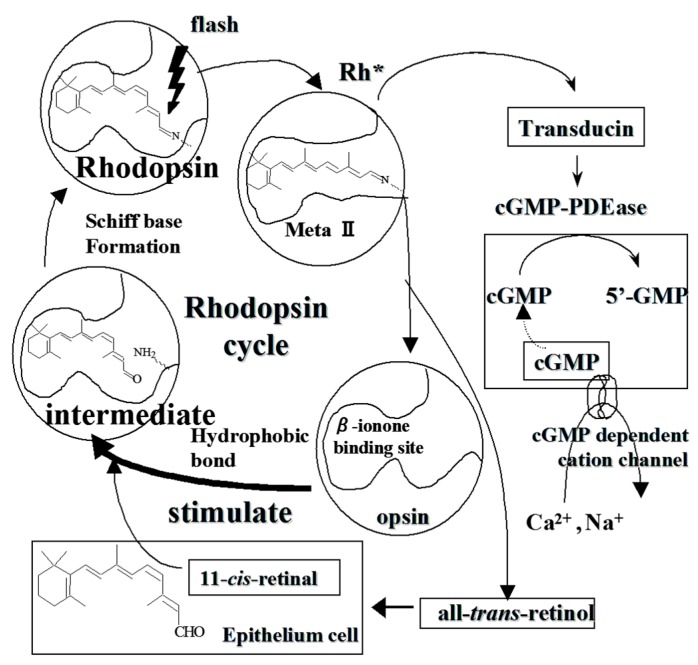
Schematic diagram of rhodopsin regeneration by anthocyanin.

In the retinal rod outer segment (ROS), G-protein-coupled receptor rhodopsin localizes on lipid bilayers on laminated discs. Following light absorption, 11-cis-retinal isomerizes to all-trans-retinal in rhodopsin chromophore. Rhodopsin subsequently changes to further conformation through some intermediates chromophore and triggers some reactions through phototransduction cascade [27]. Metarhodopsin II is one of intermediate in this cascade, which activates more than 500 transducin molecules per seconds to amplify photo signals [28,29]. Thereafter, transducin activates cGMP phosphodiesterase to hydrolyze cGMP that results in closure of cation channels and subsequent cell hyperpolarization. All components of the phototransduction pathway recover their original inactive state after light activation. Light-activated rhodopsin is inactivated by phosphorylation and is then dephosphorylated before returning to a state to opsin, which is the protein moiety of rhodopsin (Figure 2). Rhodopsin is regenerated upon binding of the opsin protein and 11-cis-retinal. ACs are proposed to affect rhodopsin regeneration [30] and bilberry extract has also been shown to stimulate rhodopsin regeneration [31].

We examined the effect of AC on rhodopsin regeneration using the four different ACs found in blackcurrants and purified 11-cis-retinal [12]. Rhodopsin regeneration was measured by 10 min reaction after 11-cis-retinal addition, with or without one of four ACs (0.1 mM) to compile a time course of rhodopsin regeneration. The kinetics of rhodopsin regeneration can be expressed by the below mentioned equation:

(1) $$11\text{-cis-retinal}\;+\;\mathrm{opsin}\;\overset{k1}{\underset{k-1}{⇄}}\mathrm{INT}\;\overset{k2}{\to }\mathrm{rhodopsin}.$$

INT is a regeneration intermediate and k_1_, k_−1_ and k_2_ are rate constants.

The velocity of equation 1 reaction with saturating 11-cis-retinal is:

v_obsd_ = k_2_ × [opsin] × [11-cis-retinal]/(K_m_ + [11-cis-retinal]), (2)

where v_obsd_ is the observed rate of rhodopsin regeneration at the actual concentration of 11-cis-retinal and K_m_ = (k_−1_ + k_2_)/k_1_.

**Figure 3:**
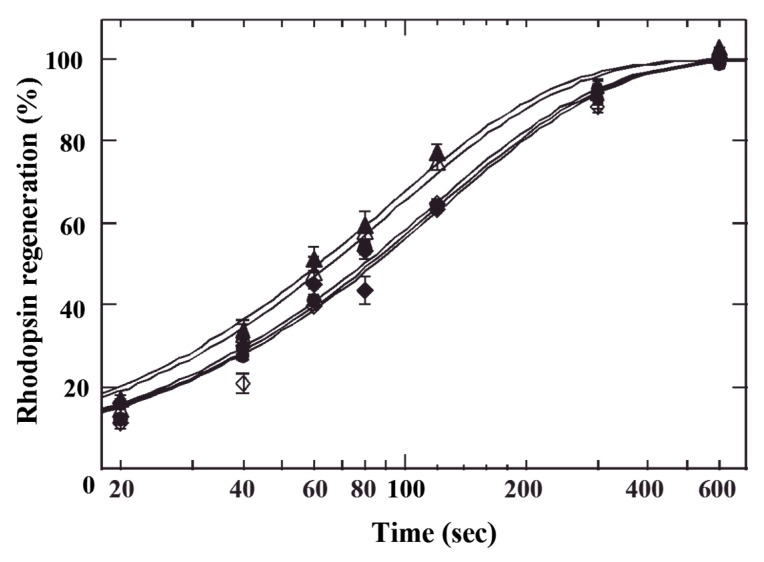
AC effects of the rhodopsin regeneration time course.  (•) control; (◇) D3G; (◆) D3R; (△) C3G; (▲) C3R. Rhodopsin regeneration was fitted to an exponential function of 1-exp (–t/τ).  Control, τ = 114 sec (R^2^ = 0.997); D3G, τ = 122 sec (R^2^ = 0.988); D3R, τ = 119 sec (R^2^ = 0.993); C3G, τ = 94 sec (R^2^ = 0.994) and C3R, τ = 89 sec (R^2^ = 0.994). Values are the mean of nine experiments, and vertical bars represent SEM.

K_m_ is a kinetic parameter that describes the regeneration of rhodopsin. D3R and D3G had no effect on regeneration of rhodopsin, whereas C3R and C3G had stimulation of regeneration (Figure 3). This result demonstrates that cyanidin mono- and diglycosides, but not D3G or D3R, accelerate rhodopsin regeneration in vitro.

**Figure 4:**
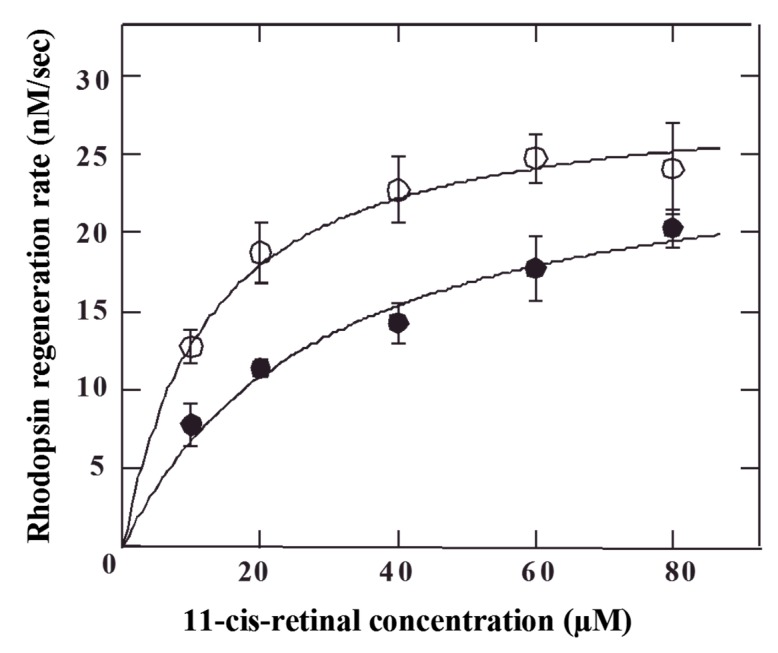
Effect of C3R on the initial rate of rhodopsin regeneration. Initial velocity of rhodopsin regeneration was determined for 40 s in the C3R (○) and control (●) at each 11-cis-retinal concentration. The data points were fitted with eq 2 to calculate rate constants in the regeneration reaction (*n* = 6, mean ± SE).

Equation (2) given above represents the observed rate of regeneration that is dependent upon [11-cis-retinal], k_1_, k_−1_ and k_2_. To estimate these rate constants, the initial rhodopsin regeneration speed was measured by v_obsd_ as a function of [11-cis-retinal]. The initial velocity of regeneration was measured for 40 s with C3R, the major cyanidin diglycoside (34.7%) in blackcurrants, at various 11-cis-retinal concentrations and the results were fitted to equation (2) to obtain k_2_ and K_m_ with or without C3R (open and closed symbols, respectively; Figure 4). C3R did not affect k_2_, but did decrease the K_m_ value by 2.4-fold compared to controls (Table 2). This decrease in K_m_ could be caused by either k_1_ increase or k_−1_ decrease, although we could not determine which change caused a K_m_ decrease. However, the results do indicate that production of a regeneration intermediate is accelerated by C3R.

Fain et al reported that, after photobleaching, opsin can still be active in the phototransduction cascade to reduce intracellular Ca^2+^ concentrations to decrease light-sensitivity of rod cells [32]. Acceleration of rhodopsin regeneration by AC might therefore promote the corresponding acceleration of rod cell light-sensitivity.

## 4. Key Effect of Anthocyanin in Ophthalmologic Field

### 4.1. Ciliary Muscle Relaxation Induced by Anthocyanins

Ciliary muscle (CM) controls lens focus, production of aqueous humor and participates in maintaining ocular pressure. CM thus has been the focus of multiple studies as a potential target for anti-glaucoma [33], myopia [34] and visual fatigue therapies [11]. To define AC recovery in transient refraction change, we examined the effect of AC on endothelin (ET)-1-induced contraction of CM to determine whether smooth muscle relaxation pathways are affected by these AC [9].

**Figure 5:**
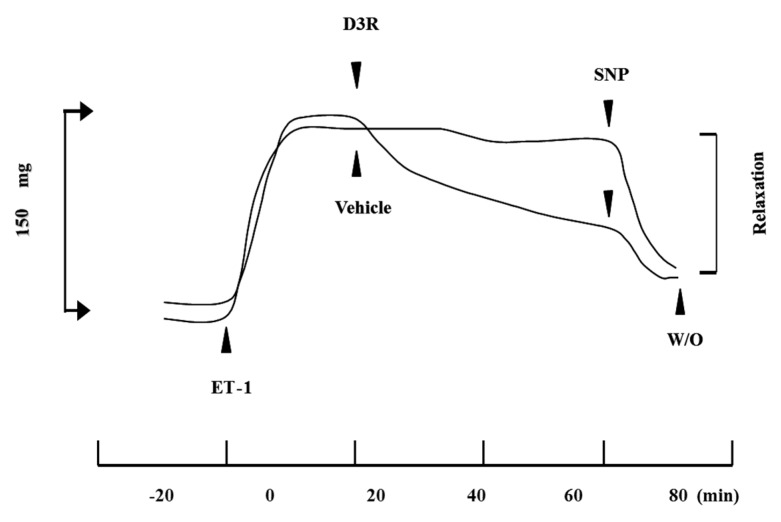
Ciliary muscle relaxation by D3R against ET-1induced contraction. 10 nM ET-1 produced a phasic contraction, followed by a tonic contraction. When the tonic contraction reached a steady level, 30 µM D3R or H_2_O (vehicle) was added and the resulting relaxation was monitored for 60 min. SNP (sodium nitroprusside) is a positive control of relaxation as an NO donor. W/O is a washout of all reagents and solution.

CM from a bovine eye was extirpated and measured of muscle tension according to a previous method [35]. After reaching stable isometric tension, CM was pre-contracted at twenty minute intervals using physiological saline solution (PSS) containing 65 mM KCl. After washing with PSS and allowing the CM to return to baseline, ET-1 was treated to stimulate muscle contraction by pharmacological concentration of 10 nM [35] and the change of isometric tension was measured using a force-displacement transducer. When the isometric tension had stabilized, a 30 µM C3R, D3R, myricetin-3-rutinoside (M3R) or quercetin-3-rutinoside (Q3R) was dropped in a single aliquot. Vehicle (distilled water) was added for the control. The relaxation within 60 min of the addition was measured to determine the relative tension (Figure 5).

**Figure 6:**
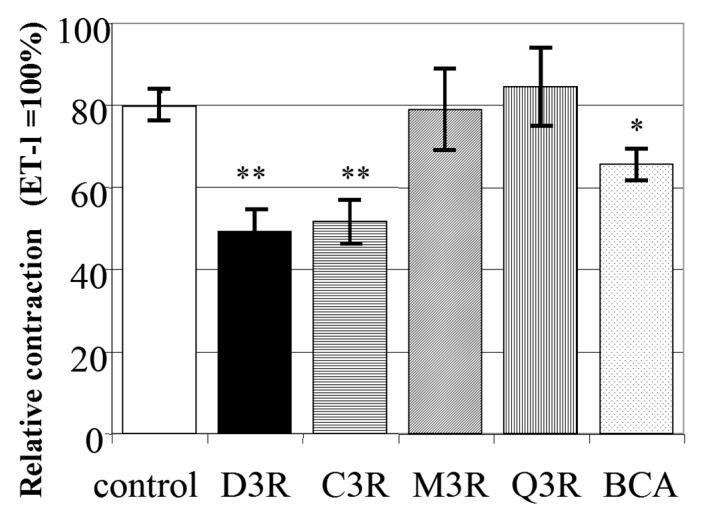
Summary of data obtained from relaxation during a 60 minute treatment of four flavonoids compared to the control. * *p* < 0.05, ** *p* < 0.01; significantly different compared to the control. The data represent the force generated with ET-1 (10 nM) set to 100%. *n* = 6, mean ± SEM.

The values for relative tension with D3R and C3R treatment had a significant difference with the control that occurred without the addition of any compound (Figure 6). Treatment with BCA (50 mg/mL) also had significant relaxation activity. Whereas Q3R and M3R, which had a similar structure with C3R and D3R, showed no relaxation activity within a 60 min treatment (Figure 6).

### 4.2. D3R Has an Inhibitory Effect against CM Contraction

CM contraction is mediated by several receptors [35,36], whereas relaxation is administrated by two mechanisms, one cAMP-dependent pathway that includes beta-adrenergic and prostaglandin receptor participated responses, and the other cAMP-independent that involves an NO involving relaxation [37]. It was reported that relaxation of bovine CM was regulated by NO similar with that of vascular smooth muscle [35]. These non-autonomic mechanisms of CM relaxation have been studied and developed both myopia and glaucoma drags [11]. Thieme reported that after unoprostone treatment a relaxation specific to ET-1 induced-contraction can be observed that is similar to that seen for pre-treatment with D3R [33]. Using this pre-treatment effect, we tried to determine whether signal transduction pathways in CM were effective using D3R pretreatment by examining contraction ratios developed as decreased % of maximum contraction (Table 3). The 0.1 mM D3R pretreatment significantly reduced contraction induced by 10 nM ET-1 from 54.9% ± 15.0% to 42.2% ± 3.2%. The influence of several inhibitors was then examined to study the mechanism of CM relaxation [33,34,38]. The beta-adrenergic blocker propranolol (0.1 mM), the K^+^ channel blocker iberiotoxin (0.1 µM) or the cyclooxygenase inhibitor indomethacin (0.1 mM) were added 20 min before the addition of D3R produced no significant change in the D3R effect on contraction induced by treatment with ET-1 (10 nM), suggesting that these pathways that are beta-adrenergic or maxi-K^+^ channels or the PGI_2_ pathway did not involve relaxation by D3R (Table 3).

Meanwhile, pretreatment for 20 min with 0.1 mM NOARG (l-N^G^-nitro arginine), the NOS inhibitor, increased the contraction ratio and reversed the D3R inhibitory effect (Table 3). The D3R contraction ratio was increased by NOARG addition and was decreased by the addition of NOARG with l-arginine. These findings imply that D3R could stimulate NO release and promote CM relaxation. Treatment with the guanylyl cyclase (GC) inhibitor ODQ (^1^H-[1,2,4] oxadiazolo [4,3-a] quinoxalin-1-one) 20 min before D3R addition increased the contraction, but ODQ alone had no effect on CM contraction. In other experiments, NO scavenger carboxy-PTIO (0.3 mM) 20 min before D3R addition increased the contraction, but carboxy-PTIO alone had a similar effect. These results suggested that D3R effect was likely caused by enhanced NO synthesis in the endothelium cells through guanylyl cyclase activation. Addition of BQ788 (0.1 µM), a selective ET_B_ receptor antagonist, augmented the contraction ratio, as did BQ788 alone. It was confirmed that D3R enhances NO synthesis that stimulates ET_B_ receptor activity in the CM.

### 4.3. Myosin Regulatory Light Chain (RLC) Phosphorylation and Cyclic

GMP production in ciliary muscle contraction of CM is activated and increased cytosolic free Ca^2+^ concentration, which saturates four Ca^2+^-binding sites of calmodulin molecules [39]. Saturated calmodulin ((Ca^2+^)4-calmodulin) activates myosin light chain kinase (MLCK) that catalyzes phosphorylation of Ser19 in motor protein myosin II at two of its 20-kDa MLC subunits (LC20) [40]. These phosphorylated reaction increases the actin-activated Mg-ATPase activity in myosin, which provides the energy of the development of force and muscle contraction [41]. MLCK can also phosphorylate LC20 at Thr18 in vitro, but only at very high (i.e., non-physiological) concentrations of the kinase [42,43]. Upon removal of Ca^2+^ from the cytosol, the CM relaxes and MLCK is deactivated whereupon myosin is dephosphorylated by MLC phosphatase, a type 1 Ser/Thr type [44].

To further examine the D3R effect against CM contraction (Table 3), we measured MLC phosphorylation in bovine CM during tension of contraction by D3R pretreatment following the addition of ET-1 (10 nM) and other agents. After treatment, the CM were quickly frozen in liquid N_2_ and then weighed before placement in a frozen slurry of 10% *wt*/*v* trichloroacetic acid in acetone containing 10 mM 1,4-dithiothreitol. We measured phosphorylated and non-phosphorylated ratio of myosin in order to evaluate phosphorylation ratio [45], and found that the ratio decreased significantly. Pretreatment with NOARG or excess l-arginine increased and decreased the ratio, respectively (Table 4).

Levels of cGMP were measured in quick-frozen CM that was treated as described above. After 10 min incubation to thaw the solution, the liquid was removed and the CM was homogenized in 60 volumes of TCA-acetone-DTT solution. Following centrifugation of the homogenate at 3000 g for 3 min, the supernatant and precipitate were removed for measurement of cGMP and myosin MLC phosphorylation concentration, respectively. The supernatant was five times extracted with four volumes of water-saturated diethyl ether to remove TCA after evaporated in a centrifugal vaporizer. The dried extract was pretreated with 10 µM 3-isobutyl-1-methylxanthine to inhibit degradation of cyclic GMP. The cGMP content was quantified using a radioimmunoassay kit [45]. Basal cGMP levels were 13.4 ± 0.6 pmol/mg tissue. ET-1 decreased these levels to 6.58 ± 0.78 pmol/mg tissue. The addition of IBMX significantly decreased ET-1 contraction (54.9% ± 3.3%), whereas the decrease mediated by D3R was 42.2% ± 3.2% where the cGMP concentration (9.39 ± 0.96 pmol/mg tissue) was increased. These changes were inhibited by the addition of 0.1 mM NOARG (5.09 ± 0.37 pmol/mg tissue).

Nathanson and McKnee demonstrated that normal human CM has highly endothelial cell NO synthase [46] and NO mediates smooth muscle relaxation by increasing cGMP production [47]. Sodium nitroprusside is a direct reagent of the NO donor, and provides rapid relaxation of smooth muscle following carbamoylcholine chloride (cholinergic agonist) or ET-1 induced contraction [48]. Furthermore, the NO synthase substrate l-canavanine has similar relaxing properties in bovine ciliary smooth muscle, particularly against ET-1 contraction compared to contraction by cholinergic agonist [11]. Thus, we hypothesized that D3R affects NO production or release that in turn leads to relaxation of ciliary smooth muscle.

### 4.4. Endothelin Receptor Binding Assay in Ciliary Muscle

To determine the ET receptor localization at which D3R mediates its effects on CM, a receptor binding assay was performed in CM and ciliary epithelium cells (CE) using [^125^I] ET-1 for saturation analysis. Specific ET binding was calculated as total binding minus non-specific binding using 125 nmol/L unlabeled ET-1. Specific binding of [^125^I] ET-1 (50 pmol/L) was replaced by the selective ET_A_ receptor antagonist BQ123 (0.1 nM to 7.81 µM) and the selective ET_B_ receptor antagonist BQ788 (0.1 nM to 7.81 µM) [9]. The [^125^I]-ET-1 binding was highly affinity in CM. Scatchard plot analysis clarified that the [^125^I]-ET-1 binding sites constituted a single population because Hill coefficients were close to unity (nH = 0.99 ± 0.02 and 1.04 ± 0.04 in CE and CM, respectively). In the CE the dissociation equilibrium constant (K_d_) and receptor density (B_max_) values were determined equal to 54.5 ± 4.6 nM (*n* = 4) and 168.4 ± 25.4 fmol/mg protein (*n* = 4), respectively. In the CM, K_d_ and B_max_ values were found 141.7 ± 18.0 nM (*n* = 4) and 357.7 ± 35.8 fmol/mg protein (*n* = 4), respectively. In the presence of BQ123, the specific [^125^I]-ET-1 binding in CM or CE was partially inhibited (by ~40%). However, binding was inhibited by BQ788, completely. The K_i_ values for BQ788 were calculated equal to 56.7 ± 10.8 pM in CE and 93.4 ± 23.3 pM in CM. Although the ET_B_ receptor sub-type predominates in both CE and CM, the binding kinetics differed.

### 4.5. Summary of Anthocyanin Effect for Ciliary Smooth Muscle Relaxation

D3R pretreatment had a preventive effect against ET-1 contraction in CM (Table 3) with a simultaneous increase in cGMP production and decrease with MLC phosphorylation. In the presence of NOARG as the NOS inhibitor, carboxy-PTIO as the NO scavenger, ODQ as the inhibitor of guanylyl cyclase or BQ788 as the ET_B_ receptor antagonist, D3R had no effect against ET-1 induced contraction (Table 3). In vascular endothelial cells, ET_B_ receptors activation by ET and resulting NO release have been suggested to promote vascular relaxation and to decrease arterial blood pressure [49]. Taken into account the previous results, it is suggested that D3R might stimulate ET_B_ receptors to release NO that results in CM relaxation. The failure of iberiotoxin as K^+^ channel inhibitor, propranolol as β-adrenoceptor antagonist and indomethacin as cyclooxygenase inhibitor to modify D3R effects on ET-1-induced relaxation indicates that ß-adrenoceptors, the PGI_2_ pathway and K^+^ channels were not involved in the mechanism by which D3R induced CM relaxation (Table 3).

**Figure 7:**
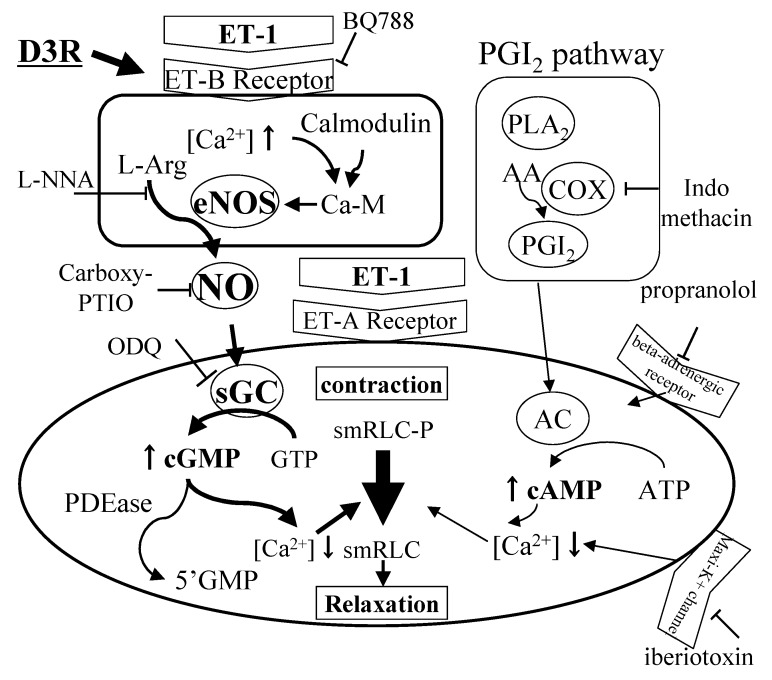
Schematic diagram of relaxation induced by D3R.

In summary, D3R relaxed in CM stimulated with ET-1 (Figure 7). Although flavonoids (Q3R and M3R) had no activity, the C-ring structure of AC did appear to be essential for the relaxation (Figure 7). These results suggest that AC could be beneficial for prevention of myopia and glaucoma.

## 5. Prevention of Myopia in a Negative Lens Fitted Chick Model

Myopia, one of the most common ocular disorders worldwide, is a serious public health issue particularly in underdeveloped areas where resources for vision correction are not readily available. A myopia experimental model in chicks, initiated by fitting a negative lens, manifests as axial length elongation in correspondence with hyperopic defocusing and is an animal model for human myopia, particularly that which develops during school-age years [50,51,52]. In the myopia model on negative lens-fitted chicks, the out of focus on the retina plays an important role in axial length elongation and changes in refractive error [53].

Based on D3R-mediated inhibition of ET-1-induced CM contraction (Table 3) and C3R stimulation of rhodopsin regeneration in frog ROS membranes (Figure 2), one theory for the onset of refractive myopia is that the CM has a continuous spasm due to excessive contraction that occurs during close up work and compromises the spasmodic refraction of crystalline lens [10]. The effect of orally administered BCA was evaluated by a myopia model on negative lens-fitted chicks [13].

**Figure 8:**
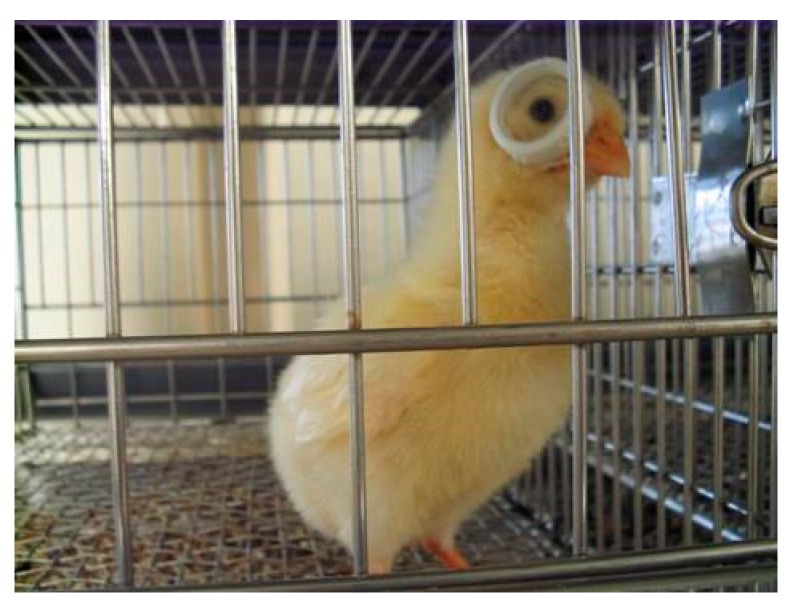
Photograph of a chick wearing a negative lens (–8D).

In this model, negative lenses (–8D) were attached by glue on the right eyes of 8-day-old white leghorn chicks while the left eyes acted as a control (Figure 8).

Axial length is the length in the eye ball from the anterior cornea surface to the bottom of the sclera [54]. Axial lengths of the chick eyes were measured with an A-scan ultrasound instrument [55] and elongation was calculated as:

Axial length elongation (mm) = axial length of right eye (mm) – axial length of left eye (mm), Ocular length elongation (mm) = ocular length of right eye (mm) – ocular length of left eye (mm).

Aqueous BCA at either 50 mg/kg BW or 100 mg/kg BW was orally administered to chicks by gavage before the application of the negative lens. Chicks then received the same BCA dose once a day for two days after fitting of a negative lens. In the control group, distilled water was administered to chicks. On the third day (72 h) after fitting of a negative lens, the axial and ocular lengths of the right eyes that had the lens increased significantly compared to those of left eyes. This result demonstrated the successful induction of myopia in chicks and was consistent with previous reports [51,52], which would allow the testing of the effects of oral BCA.

**Figure 9:**
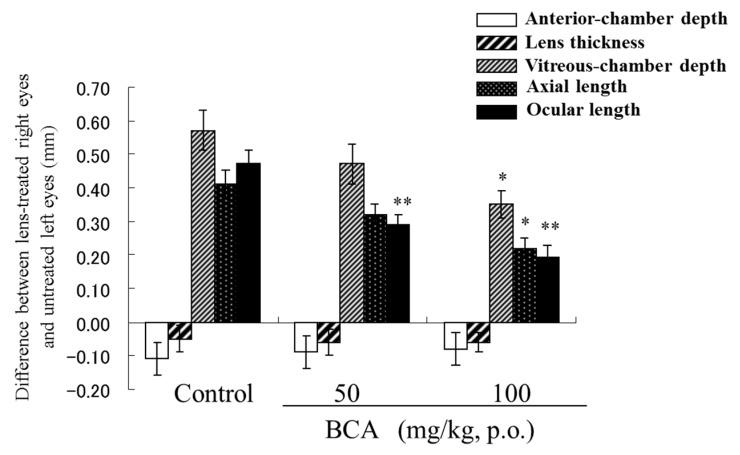
The blackcurrant anthocyanin (BCA) effect on enlargement of each eye component dimensions after application of a negative lens (–8D) in chicks. *n* = 12, mean ± SEM. paired *t*-test (intra-group) or Dunnett's multiple comparison test (inter-group). * *p* < 0.05, and ** *p* < 0.01; vs. control.

The increases in axial and ocular length caused by wearing a negative lens were significantly inhibited by orally administered BCA. The higher dose of BCA (100 mg/kg) significantly inhibited axial length elongation compared to the distilled water control (0.22 ± 0.033 mm vs. 0.41 ± 0.042 mm; Figure 9). A lower BCA (50 mg/kg) dose was also associated with a reduction in axial length elongation (0.32 ± 0.033 mm). However, the ocular length of birds fed either the dosage of BCA was significantly shorter than that of control birds. Detailed methods and results for this study are described by Iida et al. [56].

Further studies are needed to clarify the inhibition mechanism of each AC on the elongation of axial and ocular lengths, and to provide insight into therapeutically effective doses of AC contained in BCA.

## 6. Dark Adaptation Study

AC treatment is known to improve night vision [5]. In World War II, British Air Force pilots consumed blueberry jam in night flights to obtain clear night vision [3]. In a systematic review of 30 clinical studies to examine the bilberry effect on vision in reduced light conditions, Canter and Ernst found that testing of psychophysical outcome parameters was weak evidence due to a lack of strict study designs (e.g., non-randomized or non-placebo controlled trials) [6].

Based on this review, they concluded that although bilberry AC appeared to improve normal night vision, there was a lack of evidence from rigorous clinical studies to support this effect. Therefore, we performed a clinical study of the effects of AC on dark adaptation. Healthy subjects (four males, eight females, average weight 60.5 kg, average age 33.3 years-old) were assigned to four groups. One group was given a placebo and three groups were given different doses of BCA (50, 25 and 12.5 mg AC/subject). This study was carried out by a double-blind, placebo-controlled, crossover study [15].

**Figure 10:**
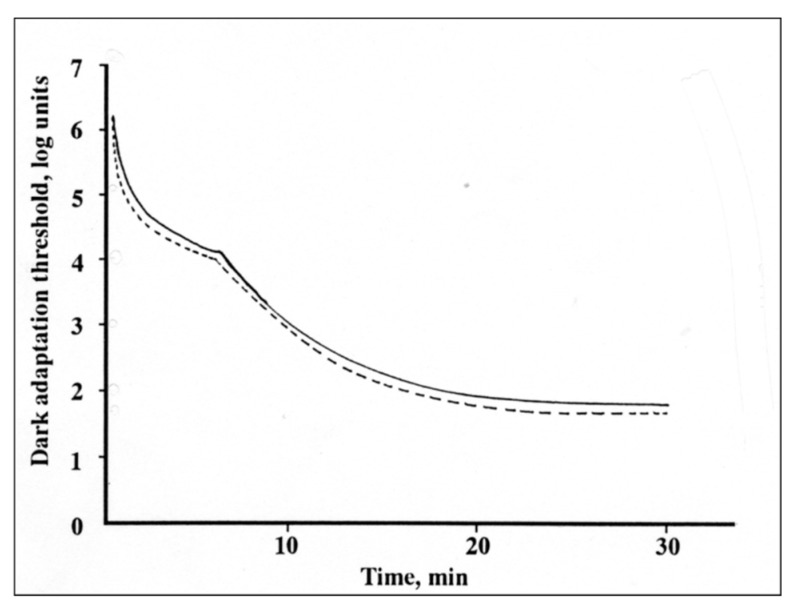
Typical dark adaptation curve before (continuous line) and after (dotted line) BCA intake.

As test samples, BCA was delivered in powders packed into capsules that were the same color as that used for placebo, which contained the same amount of sucrose. Dark adaptation values were shown as the visual threshold after 30 min of dark adaptation compared before and 2 h after intake of each test drink. Each subject was tested four times with 1 week between each test. The dark adaptation threshold was measured binocularly using a Goldmann-Weekers Adaptometer after increasing the intensity of light until the subject recognized the bars and answered its correct direction. A typical profile of dark adaptation curve by one subject is shown in Figure 10.

Comparison of mean and standard deviations for the bottom of dark adaptation threshold by log unit (log asb) at two hours after BCA intake in the four groups were shown in Table 5. Dark adaptation value in placebo group was 2.018 ± 0.218. The values in AC intake groups were 2.004 ± 0.195 (AC, 12.5 mg/subject), 1.980 ± 0.197 (AC, 25 mg/subject) and 1.923 ± 0.167 (AC, 50 mg/subject), indicating an AC dose dependency (Table 5). At an AC dose of 50 mg/subject, a significant difference (*p* = 0.014) was observed in threshold values after intake compared to placebo. Comparison of four AC dose groups before and after intake showed a significant difference (*p* = 0.011) for the 50 mg/subject AC dose (Table 5).

## 7. Transient Refractive Alteration Study

Accommodative alterations, or pseudo myopia following long-term near visual task, is thought to be the physiological parameter of eye fatigue [57]. The effects of BCA on transient refractive changes and asthenopia were tested in 21 healthy subjects (mean age, 20.9 years-old; range, 20–25 years). The study was a double-masked, randomized, crossover design, with subjects divided to either a BCA group or a placebo group. Each subject was tested at one-week intervals between BCA intake. The subjects were given 200 mL of juice containing AC (50 mg of AC/subject) or placebo. Subjects ate food 2 h before the visual task, and visual fatigue was compared before and after intake. The experimental near visual task was a modification of the Kraepelin test method [58]. The task using simple computer calculation was performed for 2 h without any rest. After completing task, the refraction values, flicker values and visual analog scale (VAS) [59] as indicators of the magnitude of asthenopia were measured.

The spherical (S) and cylindrical (C) refraction value of eye was first measured using an autorefractometer. The refraction value was evaluated in terms of the spherical equivalent of (S + C/2). The flicker value was next determined using critical flicker fusion (CFF) that was measured using a Flicker 501. Refraction values in dominant eye were summarized in Table 6. In the placebo trial, refraction values after the visual task showed a remarkable decrease that had borderline statistical significance (from −0.384 ± 0.536 to −0.503 ± 0.579, *p* = 0.064). Comparison of refraction values before and after BCA intake showed that the mean values were similar. In contrast, the mean changes of BCA and placebo were −0.030 ± 0.252 D and 0.119 ± 0.278 D, respectively. This difference was statistically significant (*p* = 0.006).

Flicker values did not show differences between BCA and placebo groups (Table 6). Mean values for the five criteria used as subjective assessment of asthenopia were higher in every group after the task, but the increase was smaller for the BCA group. Furthermore, there was a significant difference in values for BCA and placebo for asthenopia symptoms at two sites, the eye and the lower back.

Another name for transient myopic shifts is pseudo myopia, which is common in young people, but now occurs frequently among elderly people as well [60]. Patients with ophthalmologic disease often consume AC and have improvement in subjective disease symptoms. Thus, the rate of AC consumption for conditions such as pseudo myopia has increased, although AC is not effective for mitigation of symptoms for all ophthalmologic disorders.

## 8. Clinical Study of AC Effects in Glaucoma Patients

Glaucoma is an intractable disease and the major reason of blindness in worldwide [61]. The increasing intraocular pressure (IOP) is the predominant factors for onset of glaucoma, although lowering elevated IOP by medication or surgical intervention is often insufficient to prevent glaucoma progression [62]. As D3R potentially activates ET_B_ receptors to promote NO release [9], we hypothesized that AC may alter ET-1 receptor-mediated ET-1 metabolism to impact the hemodynamics of retinal blood circulation and in turn alter blood flow at optic nerve heads.

In a clinical study, thirty consecutive hospital visit patients (age range, 51–80; mean age 66.7 ± 6.9 years-old, nine males and 21 females) with normal tension glaucoma (NTG) received 50 mg orally administered BCA once daily for 6 months [63]. Blood flow at the neuroretinal rim of the optic nerve head and peripapillary retina was evaluated using a scanning laser Doppler flow meter both before and after 6 months of administration [64]. The IOP for every patient almost remained stable in BCA group during the test period. However, ET-l concentrations in plasma had significantly increased from 3.27 ± 1.67 (mean ± S.D.) pg/mL to 4.10 ± 2.14 pg/mL before and just after the administration period, respectively (*p* < 0.05). After administration, the blood flow of the superior (Sup) and inferior (Inf) temporal neuroretinal rim of the optic nerve head and peripapillary retina in both eyes were significantly altered (Table 7).

Neuroretinal rim and peripapillary retinal blood flow, which are correlated with degradation of visual field [65,66], were both significantly reduced in patients with NTG compared to healthy individuals.

Ohguro et al. also conducted a randomized, placebo-controlled, double-masked 24-month study on the effects of BCA treatment on progression in 38 open-angle glaucoma (OAG) patients treated with anti-glaucoma drops [16]. They found that BCA treatment (50 mg/day, *n* = 19) reduced the degradation of the visual field and elevations in retinal blood flow of OAG. The BCA group had significantly better ocular blood circulation relative to both baseline values and placebo-treated patients as measured by the Humphrey visual field mean deviation in the eye (*p* = 0.039). However, other systemic and ocular conditions showed no significant changes in both groups.

In another study of OAG patients, Ohguro et al. found that the concentration of serum ET-1 among OAG patients was significantly lower than that in healthy subjects (*p* < 0.05). After BCA intake, ET-1 concentration in serum increased to levels that were similar to those of healthy subjects, whereas ET-1 values for placebo-treated patients remained lower [17]. The results of these clinical studies suggest that AC intake might be a low risk and effective option for neuroprotective treatment with OAG and NTG patients.

## 9. Conclusions

Antioxidative flavonoids such as AC are abundant in fruits and vegetables. The therapeutic application of AC by the ophthalmological field has shown potential for improving eye health. In this review, we summarized several studies showing that AC has specific effects on rhodopsin regeneration and smooth muscle relaxation in vitro. In studies in vivo, AC was present in eye tissues and improved blood circulation. These findings were also reflected in clinical trials of BCA to improve vision health and together these studies suggest that BCA could be an effective nutraceutical product for the treatment and prevention of an optical disorder.

However, the use of AC in the pharmaceutical field is limited by its low bioavailability and instability at neutral pH. Moreover, AC degradation products were difficult to be tracted and evaluated in in vitro studies. Further studies are needed to determine the structure of AC degradation products at neutral pH and confirm its functionality at physiological conditions.

## Figures and Tables

**Table 1:** The ocular tissue distribution of AC at 1 h post-intraperitoneal administration in rats and at 30 min post-intravenous administration in rabbits. Mean ± SE, *n* = 5 in rats, and *n* = 3 in rabbits.

Ocular Tissues or Body Fluid	Intraperitoneal Administration in Rat		Intravenously Administration in Rabb	
	AC of Tissue (μg/g Tissue)	Distribution Ratio (%)	AC of Tissue (μg/g Tissue)	Distribution Ratio (%)
Aqueous humor	6.72	0.88	1.19 ± 0.21	10.54
Cornea	20.62	3.67	0.55 ± 0.05	4.89
Sclera	245.04	89.09	3.02 ± 0.09	26.73
Choroid			3.00 ± 0.06	26.57
Ciliary body	12.93	1.39	2.04 ± 0.28	18.07
Iris			1.11 ± 0.08	9.81
Retina	6.89	4.76	0.27 ± 0.02	2.41
Vitreous	0.60	0.14	0.11 ± 0.02	0.98
Lens	0.36	0.06	0.00 ± 0.00	0.00
Plasma *	2.30 ± 0.76		12.42 ± 1.25	

*: μg/mL.

**Table 2:** Kinetic parameters of rhodopsin regeneration.

	Control *	+C3R *
k_2_ (s^−1^)	1.0 × 10^−2^	1.1 × 10^−2^
km (M)	2.6 × 10^−5^	1.1 × 10^−5^

* R^2^ = 0.989 (control); 0.995 (+C3R).

**Table 3:** Comparison of contraction of the ciliary muscle by ET-1 (10 nM) after treatment with various reagents.

Treatment	Concentration	*n*	Contraction (%) (Average ± SE)
control		21	54.9 ± 3.3 ^a^
D3R	10 mM	21	42.2 ± 3.2
D3R + NOARG	10 mM + 10 mM	12	63.7 ± 7.1 ^a^
D3R + NOARG+l-Arg	10 mM + 10 mM + 10 mM	12	42.7 ± 4.2
Carboxy-PTIO	30 mM	10	54.4 ± 2.1
D3R + Carboxy-PTIO	10 mM + 30 mM	10	55.2 ± 3.2 ^a^
ODQ	10 mM	11	56.1 ± 5.4
D3R + ODQ	10 mM + 10 mM	11	58.5 ± 5.5 ^a^
BQ788	10 μM	12	81.0 ± 8.2
D3R + BQ788	10 mM + 10 μM	12	73.4 ± 9.6 ^a^
propranolol	10 mM	9	61.3 ± 3.6
D3R + propranolol	10 mM + 10 mM	8	48.7 ± 3.4 ^b^
iberiotoxin	10 μM	12	64.6 ± 3.0
D3R + iberiotoxin	10 mM + 10 μM	12	45.7 ± 4.8 ^c^
indomethacin	10 mM	12	70.3 ± 5.3
D3R + indomethacin	10 mM + 10 mM	12	52.4 ± 5.2 ^d^

^a^ Significant difference at *p* < 0.05 vs. D3R treatment; ^b^ Significant difference at *p* < 0.05 vs. propranolol treatment; ^c^ Significant difference at *p* < 0.05 vs. iberiotoxin treatment; ^d^ Significant difference at *p* < 0.05 vs. indomethacin treatment.

**Table 4:** Comparison between ET-1 induced ciliary muscle contraction ratio and phosphorylated MLC ratio.

Treatment	Concentration	Contraction (%) (Average ± SE)	Phosphorylated-Ratio (%) (Average ± SE)
control		54.9 ± 3.3 ^a^	47.2 ± 13.1 ^a^
D3R	10 mM	42.2 ± 3.2	36.0 ± 13.6
D3R + NOARG	10 mM + 10 mM	63.7 ± 7.1 ^a^	50.1 ± 4.2 ^a^
D3R + NOARG + l-Arg	10 mM + 10 mM+ 10 mM	42.7 ± 4.2 ^b^	38.2 ± 3.7 ^c^

^a^ Significant difference at *p* < 0.05 vs. D3R treatment; ^b^ Significant difference at *p* < 0.05 vs. D3R + NOARG; ^c^ Significant difference at *p* < 0.01 vs. D3R + NOARG.

**Table 5:** Dark adaptation threshold changes before and after intake of the test drink.

Dose of BCA	Dark Adaptation Value, Mean ± SD of Log Asb; (*p* value) ^(1)^			
mg/subject	Before Intake	After Intake	Change	*p* value ^(2)^
0 (placebo)	2.056 ± 0.209 (1.000)	2.018 ± 0.218 (1.000)	−0.038 ± 0.106 (1.000)	0.244
12.5	2.026 ± 0.147 (0.457)	2.004 ± 0.195 (0.457)	−0.023 ± 0.138 (0.733)	0.583
25	2.016 ± 0.170 (0.234)	1.980 ± 0.197 (0.264)	−0.037 ± 0.112 (0.983)	0.28
50	2.038 ± 0.186 (0.686)	1.923 ± 0.167 (0.014)	−0.115 ± 0.131 (0.171)	0.011

^(1)^ The results of statistical analysis, independently carried out in each vertical row, are shown in parentheses. ^(2)^ Statistical *p* value for “after intake” vs. “before intake” in each horizontal row.

**Table 6:** Refraction values in dominant eye, flicker values, assessment of subjective asthenopia and changes in transient refractive alteration measured before and after a visual task in subjects orally administered BCA or placebo ^1^.

Item & Statement	BCA			Placebo		
	Before	After ^2^	Change ^2^	Before	After ^2^	Change ^2^
Refraction values	−0.432 ± 0.602	−0.402 ± 0.643 ^a^	−0.030 ± 0.252 ^(a),c^	−0.384 ± 0.536	−0.503 ± 0.579 ^b^	0.119 ± 0.278 ^(b), (c)^
Flicker value, Hz	34.95 ± 3.16	34.39 ± 3.51	0.56 ± 1.15	34.72 ± 2.99	34.13 ± 2.90	0.59 ± 1.22
Asthenopia symptoms (VAS mm)						
head & neck	12.70 ± 13.45	40.08 ± 24.86	27.38 ± 18.39	8.34 ± 11.87	44.09 ± 26.09	35.75 ± 24.96
arm	10.21 ± 17.82	36.15 ± 25.86	25.94 ± 29.61	4.32 ± 6.27	41.76 ± 29.33	37.44 ± 28.43
eye	14.72 ± 15.55	47.31 ± 24.72	32.59 ± 18.94 ^(a)^	14.59 ± 17.98	56.72 ± 25.24	42.14 ± 19.52 ^(b)^
shoulder	15.12 ± 15.65	49.66 ± 27.97	34.54 ± 25.75	10.95 ± 17.42	54.31 ± 29.31	43.36 ± 30.91
low back	10.63 ±15.92	29.79 ± 27.15 ^a^	19.16 ± 22.74 ^(a)^	7.35 ± 9.25	42.83 ± 33.55 ^b^	35.48 ± 30.87 ^(b)^

^1^ Expressed as mean ± SD. ^2^ Statistical analysis comparing the values after the test and the change in values was independently carried out in each horizontal row. ^a, b, c, (a), (b), (c)^ Values with different superscript letters are significantly different (*p* < 0.05).

**Table 7:** Retinal blood flow of the superior temporal rim/retina and inferior temporal/retina after consumption of anthocyanins for 6 months in the left eye (LE) and right eye (RE).

Treatment	Before (Average ± SE)	After 6 Month (Average ± SE)
Sup. temp. rim (RE)	507.7 ± 174.3	638.6 ^a^ ± 191.2
Inf. temp. rim (RE)	393.6 ± 138.0	582.2 ^b^ ± 177.8
Sup. temp. retina (RE)	457.6 ± 140.6	595.1 ^b^ ± 171.5
Inf. Temp. retina (RE)	377.0 ± 80.5	519.1 ^b^ ± 130.0
Sup. temp. rim (LE)	442.4 ± 214.3	662.4 ^b^ ± 185.3
Inf. temp. rim (LE)	466.5 ± 216.3	653.7 ^b^ ± 260.9
Sup. temp. retina (LE)	375.0 ± 75.9	442.2 ^b^ ± 80.1
inf. temp. rim (LE)	444.9 ± 100.9	546.9 ^a^ ± 185.8

^a^ Significant difference at *p* < 0.05 vs. before intake; ^b^ Significant difference at *p* < 0.01 vs. before intake.
